# Allograft nerve repair to prevent sensorimotor loss after nerve sheath tumor resection

**DOI:** 10.3171/2022.10.FOCVID22101

**Published:** 2023-01-01

**Authors:** Stephen P. Miranda, Jessica Nguyen, Ben J. Gu, Zarina S. Ali, Eric L. Zager

**Affiliations:** Department of Neurosurgery, Hospital of the University of Pennsylvania, Philadelphia, Pennsylvania

**Keywords:** nerve allograft, nerve sheath tumor, schwannoma, nerve repair

## Abstract

Acellularized nerve allografts (ANAs) have been developed as substitutes for nerve autograft to promote nerve regeneration after surgical repair. In this video, the authors demonstrate operative techniques for using ANAs to repair potentially functional nerve fascicles during tumor resection. A 67-year-old female with schwannomatosis requested resection of a painful enlarging mass of the left ulnar nerve proximal to the elbow. During surgery, neuromonitoring suggested that fascicles entering the tumor could be functional. Therefore, nerve allograft was used to repair the transected fascicles. The patient recovered with full strength and sensation in the ulnar distribution, with resolution of her preoperative symptoms.

The video can be found here: https://stream.cadmore.media/r10.3171/2022.10.FOCVID22101

## Transcript

In this video, we demonstrate operative techniques for using nerve allograft to repair potentially functional nerve fascicles during nerve sheath tumor resection.

### 0:29 Background.

Acellularized nerve allografts (ANAs) have been developed as a promising substitute for nerve autograft.^1^ After fresh transplanted nerve allografts were first shown to be as efficacious as autologous nerve for short gaps, decellularization techniques have since been developed to avoid the need for immunosuppression during host axonal regeneration.^2^ These processing techniques allow the allografts to retain the extracellular matrix and endoneurial architecture of the native nerve, which promote cell migration, nerve fiber elongation, and axonal outgrowth.^2^ ANAs are now widely available and approved for clinical application in a variety of sizes, to facilitate nerve repair techniques for sensorimotor deficits.^3–5^ In this case, allograft nerve was used to repair potentially functional nerve fascicles after nerve sheath tumor resection.

### 1:17 Patient Presentation.

This 67-year-old right-handed woman had a history of schwannomatosis with multiple prior resections of painful schwannomas. She presented with an enlarging painful mass in the medial aspect of her left arm above the elbow. The tenderness above her elbow began about a year prior, with pain radiating into the fourth and fifth digits of her left hand. The mass was tender to touch, and she reported "electric shocks" whenever she bumped into things. She denied any numbness, paresthesias, weakness, or loss of coordination. On examination, the patient had full strength and sensation with a positive Tinel’s sign at the site of the mass. Imaging studies suggested a nerve sheath tumor arising from the ulnar nerve measuring 1.8 × 1.3 × 1.5 cm, located in the left medial arm.

### 2:03 Surgical Intervention.

After a period of observation, her symptoms eventually became disabling, and the patient requested neurosurgical intervention. Informed consent was obtained for left ulnar nerve exploration, internal neurolysis, resection of nerve sheath tumor, with possible fascicular nerve repair using allograft nerve, if needed. The patient also provided informed consent for intraoperative recording of photographs and videos.

The patient was taken to the operating room, and after general endotracheal anesthesia was induced, no further muscle relaxant was used, so that neuromonitoring could be performed. The patient was positioned supine with the left arm abducted and externally rotated on arm boards. Neuromonitoring leads were placed after the entire left upper extremity was sterilely prepped and draped in the usual fashion. A linear incision was then marked overlying the palpable mass in line with the ulnar nerve in the medial arm. The skin was infiltrated with local anesthetic, and the incision was opened with a No. 15 blade. Dissection then proceeded through the subcutaneous tissue and fascia to expose the ulnar nerve and the nerve sheath tumor. Overlying branches of the medial antebrachial cutaneous nerve were preserved. The tumor had the typical appearance of a benign nerve sheath tumor. The ulnar nerve and its fascicles overlying the tumor were then stimulated with the assistance of the neuromonitoring team. An electrically "silent" zone on the posterior aspect of the tumor was then identified and opened sharply so that the true capsule of the tumor could be gently dissected. This allowed us to preserve the fascicles of the ulnar nerve, which were functional. There were two small fascicles entering the tumor, which stimulated at higher current levels. Because they clearly entered the tumor, they could not be preserved during tumor resection. There were three tiny fascicles exiting the tumor, which also had higher threshold for stimulation. Therefore, these fascicles were also divided in order to allow complete resection of the tumor en bloc. There was no motor response during sectioning. The mass was sent for permanent pathology. We then elected to place a 2- to 3-mm acellular allograft nerve segment for microsurgical repair of the fascicles that were divided. We cut the allograft to the gap, which measured approximately 2 cm in length. The operating microscope was brought into the field, and microsurgical technique was used for the nerve repair. A single suture of 8-0 nylon was placed at each repair site, having grouped the two fascicles proximally and the three small fascicles distally to have excellent coaptation with the allograft. The wound was then irrigated with saline, and meticulous hemostasis was obtained. The repair sites were covered with fibrin glue.

### 5:34 Closure.

The incision was closed in anatomical layers using interrupted 3-0 absorbable sutures, and a running 4-0 subcuticular suture with adhesive strips for the skin closure. A dry sterile dressing was placed with a compressive wrap to aid in hemostasis, which would be removed on postoperative day 2. The patient was extubated and taken to the recovery room in satisfactory condition. She had full strength and sensation postoperatively in the ulnar distribution. Pathology revealed WHO grade I schwannoma.

## Disclosures

The authors report no conflict of interest concerning the materials or methods used in this study or the findings specified in this publication.

## Author Contributions

Primary surgeon: Zager. Assistant surgeon: Miranda. Editing and drafting the video and abstract: Miranda, Nguyen, Ali. Critically revising the work: Miranda, Gu, Ali, Zager. Reviewed submitted version of the work: Miranda, Gu, Ali, Zager. Approved the final version of the work on behalf of all authors: Miranda. Supervision: Ali, Zager.

## Supplemental Information

### Patient Informed Consent

The necessary patient informed consent was obtained in this study.
